# Bone turnover in arginine vasopressin deficiency: a comparative study with primary polydipsia and healthy controls

**DOI:** 10.1210/clinem/dgag001

**Published:** 2026-01-07

**Authors:** Emanuele Varaldo, Sven Lustenberger, Cihan Atila, Sophie Monnerat, Laura Potasso, Mirjam Christ-Crain

**Affiliations:** Department of Endocrinology, Diabetology and Metabolism, University Hospital Basel, Basel 4031, Switzerland; Department of Clinical Research, University Hospital Basel, University of Basel, Basel 4031, Switzerland; Division of Endocrinology, Diabetology and Metabolism, Department of Medical Sciences, University of Turin, Turin 10126, Italy; Department of Endocrinology, Diabetology and Metabolism, University Hospital Basel, Basel 4031, Switzerland; Department of Clinical Research, University Hospital Basel, University of Basel, Basel 4031, Switzerland; Department of Endocrinology, Diabetology and Metabolism, University Hospital Basel, Basel 4031, Switzerland; Department of Clinical Research, University Hospital Basel, University of Basel, Basel 4031, Switzerland; Department of Endocrinology, Diabetology and Metabolism, University Hospital Basel, Basel 4031, Switzerland; Department of Clinical Research, University Hospital Basel, University of Basel, Basel 4031, Switzerland; Department of Endocrinology, Diabetology and Metabolism, University Hospital Basel, Basel 4031, Switzerland; Department of Clinical Research, University Hospital Basel, University of Basel, Basel 4031, Switzerland; Department of Endocrinology, Diabetology and Metabolism, University Hospital Basel, Basel 4031, Switzerland; Department of Clinical Research, University Hospital Basel, University of Basel, Basel 4031, Switzerland

**Keywords:** central diabetes insipidus, AVP deficiency, oxytocin, CTX, P1NP, osteoporosis

## Abstract

**Context:**

Arginine vasopressin (AVP) and oxytocin are neurohormones with opposing effects on bone in preclinical models, while their relevance in humans remains uncertain. Data on bone metabolism in individuals lacking AVP release—and possibly also oxytocin—such as patients with AVP deficiency (AVP-D, also known as central diabetes insipidus) remain scarce, although the limited available evidence suggests a potentially deleterious effect.

**Objective:**

This study aimed to evaluate bone turnover markers in patients with AVP-D compared to individuals with primary polydipsia (PP) and healthy controls (HC).

**Methods:**

This was a secondary analysis of the prospective URANOS Trial (NCT05890690) conducted from June 2023 to June 2024. After excluding patients on chronic steroid therapy (except budesonide and replacement therapy), prior osteoporotic fractures, long-term antiresorptive treatment, or medications known to adversely affect bone metabolism (eg, aromatase inhibitors), 46 participants were included (HC = 22, AVP-D = 11, PP = 13). All individuals were assessed for bone resorption (C-terminal telopeptide of type I collagen [CTX]) and bone formation (N-terminal propeptide of type I collagen [P1NP]) markers, as well as 25OH-vitamin D, serum calcium, and phosphate. The bone formation index (defined as P1NP/CTX ratio) was calculated.

**Results:**

Serum calcium, phosphate, and P1NP levels were comparable across groups, whereas 25OH-vitamin D concentrations were lower in patients with AVP-D than in HC (*P* = .031). Median CTX levels were lower in AVP-D (0.373 [0.288-0.513] ng/mL) than in HC (0.592 [0.427-0.729] ng/mL, *P* = .021), with no difference between PP (0.514 [0.411-0.618] ng/mL) and the other groups. The bone formation index was higher in AVP-D than in HC (*P* = .036), whereas no difference was observed between PP and either group. In the multivariable linear regression model adjusted for confounders, CTX was lower in AVP-D compared to HC (−0.183; 95% CI, −0.352 to −0.013; *P* = .036), with no difference compared to PP.

**Conclusion:**

Patients with AVP-D showed reduced CTX levels and consequently an increased P1NP/CTX ratio compared to HC, whereas no difference was observed between patients with PP and both groups. Overall, these findings do not support major bone metabolic alterations with detrimental effects in AVP-D.

Arginine vasopressin (AVP) and oxytocin (OXT) are nonapeptide neurohormones synthesized in the supraoptic and paraventricular nuclei of the hypothalamus, from which they are released into the bloodstream via axonal projections to the posterior pituitary (1).

Beyond its primary role in maintaining water-electrolyte homeostasis through its action on the renal collecting duct, AVP also has receptors expressed in bone tissue. Specifically, both the V1a and V2 receptors are present on osteoclasts and osteoblasts, which also express OXT receptors (2). Preclinical studies in mice have reported an opposing effect of these 2 hormones: overall, AVP is attributed with a negative regulatory role in bone metabolism, reducing bone formation while simultaneously enhancing bone resorption (2-4). In this context, the predominant effect appears to be mediated primarily by the V1a receptor rather than the V2 receptor because the specific V2 receptor inhibitor tolvaptan has not been reported to affect bone formation or bone mass (5). In contrast, OXT exerts an anabolic effect by promoting osteoblast differentiation and function (2, 4, 5).

Although both hormones exert well-established effects on osteoblasts and osteoclasts in murine models, their relevance in humans remains uncertain. Specifically, data on the impact of an AVP deficient state on bone metabolism remain limited and only a few studies are available. Importantly, it was recently shown that patients with AVP deficiency (AVP-D, also known as central diabetes insipidus) are not only deficient in AVP but also likely exhibit a concomitant deficiency of OXT, the second hormone produced by the same hypothalamic nuclei (6-8). In their analysis published in 1998, Pivonello et al reported that patients with AVP-D exhibited significantly lower osteocalcin levels (at the time the most reliable marker of bone formation) compared to healthy controls, despite similar urinary cross-linked N-telopeptide of type I collagen (NTX) concentrations (at the time the most used marker for bone resorption) (9). Furthermore, the same research group later demonstrated that a short-term, 6-month treatment with alendronate led to a significant reduction in NTX levels from baseline, accompanied by a significant increase in bone mineral density (BMD) at the lumbar spine and femoral neck (10). More recently, in 2021, Aulinas et al also reported that patients with AVP-D and reduced fasting plasma OXT levels had lower BMD and less favorable hip geometry than those with higher fasting OXT levels. However, this study was based only on bone characteristics and no data on bone metabolism markers were available (11).

Given the substantial improvement over time in the reliability of bone formation and resorption markers, and the limited data currently available, the objective of our study was to evaluate bone metabolism in patients with AVP-D. Specifically, we aimed to compare bone turnover markers in these patients not only with healthy controls (HC), but also with individuals with primary polydipsia (PP), a group expected to exhibit fluctuating suppression of AVP levels while maintaining presumably preserved OXT secretion. To our knowledge, this is the first study to investigate bone metabolism in this setting using the most accurate biochemical indicators currently available, namely serum N-terminal propeptide of type I collagen (P1NP) and serum C-terminal telopeptide of type I collagen (CTX), which reflect bone formation and bone resorption, respectively.

## Materials and methods

This was a preplanned secondary analysis of a prospective study involving patients with either AVP-D or PP and HC undergoing a novel copeptin stimulation test based on urea administration. Full details regarding the study rationale, design, and statistical analyses have been published elsewhere (12).

In brief, in the first part of the study, healthy adults (≥18 years) with no regular medication other than hormonal contraception and no history of polyuria-polydipsia syndrome (defined as urine output >40 mL/kg/day and/or fluid intake >3 L/day) underwent 2 study visits, separated by a washout period of at least 3 days. During each visit, participants received either oral urea (OMANDA AG, CH-3072 Ostermundigen, administered at a dose of 0.5 g/kg body weight dissolved in 200 mL of water; range 30-45 g) or placebo.

In the second part of the study, adult patients (≥18 years) with a confirmed diagnosis of AVP-D or PP—based on a prior water deprivation test, hypertonic saline infusion test, or arginine infusion test—underwent a single study visit. During this visit, they received an open-label urea drink prepared identically to the that used for healthy adults.

All participants presented in the morning after overnight fasting and a 2-hour period of fluid restriction. Before the visit, they were instructed to avoid physical exercise and alcohol for 24 hours, and to abstain from smoking on the test day. Patients on desmopressin therapy were instructed to withhold the drug for at least 12 hours before testing, whereas those on cortisol replacement therapy were advised to take their regular stress-dose regimen before the visit. Exclusion criteria included pregnancy, breastfeeding, or known allergies to any component of the study drink. The local ethics committee approved the protocol (EKNZ 2023-00751), and written informed consent was obtained from all participants before any study procedures. The study was conducted in accordance with the Declaration of Helsinki and was registered on ClinicalTrials.gov (NCT05890690).

At time 0—immediately before urea or placebo administration during the first visit for HC, and at the same time point during the single visit for patients with AVP-D or PP—a blood sample was collected for CTX, P1NP, calcium, phosphate and 25OH-vitamin D. Patients receiving chronic steroid treatment (with the exception of budesonide (13-15) and corticosteroid replacement therapy) or drugs known to adversely affect bone metabolism (eg, aromatase inhibitors), those with a history of previous fractures, and those undergoing chronic treatment with bone antiresorptive medications were excluded from the current analysis.

### Laboratory measurements

Blood samples were drawn and immediately centrifuged to extract serum, which was stored at −80 °C until analysis. CTX, P1NP, and 25OH-vitamin D were analyzed on the IDS-iSYS Multi-Discipline automated analyzer (IDS iSYS, Immunodiagnostics Systems, Boldon, UK). The assays are based on chemiluminescence technology. The intra- and inter-assay coefficient of variation was 2.4% to 4.2% for CTX, 1.3% to 2.4% for P1NP, and 3.8% to 6.8% for 25OH-vitamin D, respectively. Bone formation index, defined as the P1NP/CTX ratio, was calculated as previously described (16, 17).

Calcium and phosphate were analyzed on the autoanalyzer Cobas c303 (Roche Diagnostics Rotkreuz, Switzerland). The intra- and inter-assay coefficient of variation was 0.5% to 0.75% for calcium and 0.62% to 0.91% for phosphate.

### Statistical analysis

Data are presented as median and interquartile range for continuous variables and as counts (n) and percentages for categorical variables. Comparisons of continuous variables were conducted using the Mann-Whitney test. Additionally, linear regression models were fitted using P1NP, CTX, and the P1NP/CTX ratio as dependent variables, with diagnosis (AVP-D or PP) as the main independent variable and HC serving as the reference group. All models were adjusted for age, sex, and smoking status. No adjustment for multiple testing was performed, and *P* values should be interpreted as descriptive measures rather than confirmatory evidence.

All statistical analyses were performed using R version 4.4.1 (R Core Team, 2024) (18), and *P* < .05 was considered statistically significant.

## Results

Out of the 48 subjects enrolled in the original study, 2 patients with AVP-D were excluded from the current analysis (1 patient because of chronic treatment with mometasone cream and another patient because of chronic treatment with exemestane). No patients were excluded because of the presence of previous fractures or concomitant therapy with antiresorptive drugs. As a result, 46 participants (HC = 22, AVP-D = 11, PP = 13) were included.

Among the participants, 2 individuals with PP and 1 with AVP-D were receiving budesonide therapy; additionally, 1 participant with PP was receiving vitamin D and calcium supplementation.

Within the AVP-D cohort, 6 patients had isolated AVP-D, whereas 5 were affected by panhypopituitarism. Of these, 2 were receiving full hormone replacement therapy, including rhGH, whereas the remaining 3 were not on rhGH therapy. All patients were on stable hormone replacement therapy. Notably, 1 woman with secondary hypogonadism, of an age compatible with physiological menopause, was not receiving gonadotropin replacement therapy.

Desmopressin therapy varied across patients: 4 were receiving intranasal administration (2 puffs), 4 were on the oral formulation (range, 0.05-0.2 mg), and 3 were treated with the sublingual formulation (range, 60-120 µg). Disease duration ranged from 3 months to 16 years and only 2 patients had a disease duration of less than 12 months. In 3 of 11 patients (27.3%), the diagnosis dated back several years, and the onset of symptoms could not be reliably recalled.

Information regarding the etiology of the disease and the current endocrinological therapy of patients with AVP deficiency is presented in Table 1, whereas detailed clinical and biochemical characteristics of the study participants are summarized in Table 2.

**Table 1:** Clinical characteristics of patients with arginine vasopressin deficiency

Patient (sex/age)	BMI (kg/m^2^)	Etiology of the disease	Duration of the disease	Desmopressin formulation	Desmopressin dose	Concomitant hormonal replacement therapy
1 (m/22)	23.0	Postneurosurgical (GH-secreting macroadenoma)	5 years	Nasal	2 puffs/day	Hydrocortisone 20 + 10 mg/day Levothyroxine 100 µg/day Testosterone undecanoate 1000 mg IM every 12 weeks
2 (m/63)	27.7	Postneurosurgical (nonfunctioning macroadenoma, FSH/LH+)	11 months	Oral	0.05 mg/day	Hydrocortisone 10 + 10 mg/day Levothyroxine 125 µg/day Testosterone undecanoate 1000 mg IM every 10 weeks
3 (f/31)	21.5	Familial form (autosomal dominant)	Several years	Sublingual	120 µg/day	—
4 (f/28)	23.4	Familial form (autosomal dominant)	Several years	Sublingual	120 µg/day	—
5 (f/26)	23.0	Idiopathic	4 years	Oral	0.2 mg/day	—
6 (f/69)	27.7	Postneurosurgical (Rathke cleft cyst)	Several years	Oral	0.1 mg/day	Hydrocortisone 10 + 10 mg/day Levothyroxine 100 µg/day
7 (f/35)	39.8	Idiopathic	3 months	Sublingual	60 µg/day	—
8 (m/33)	22.9	Hypophysitis	16 years	Nasal	2 puffs/day	—
9 (f/37)	26.4	Idiopathic	7 years	Nasal	2 puffs/day	—
10 (m/23)	24.6	Postneurosurgical (nonfunctioning macroadenoma)	1.5 years	Oral	0.2 mg/day	Hydrocortisone 10 + 10 mg/day Levothyroxine 50 µg/day Human chorionic gonadotropin 1000 IU SC, 3 times/week rhGH 0.3 mg/day SC
11 (m/46)	34.5	Postneurosurgical (craniopharyngioma)	12 years	Nasal	2 puffs/day	Hydrocortisone 20 + 10 mg/day Levothyroxine 150 µg/day Testosterone undecanoate 1000 mg IM every 10 weeks rhGH 0.3 mg/day SC

Abbreviations: BMI, body mass index; f, female; IM, intramuscular; m, male; SC, subcutaneous.

**Table 2:** Clinical and biochemical characteristics of healthy adults and patients with arginine vasopressin (AVP) deficiency and primary polydipsia

	Healthy adults (n = 22)	Patients with AVP deficiency*^a^* (n = 11)	Patients with primary polydipsia (n = 13)
**Characteristics**			
Female (sex)	12 (55)	6 (55)	11 (85)
Age (years)	27 [26-32]	33 [27-42]	30 [25-59]
BMI (kg/m^2^)	23.2 [21.6-25.8]	24.6 [23.0-27.7]	23.7 [20.6-27.1]
Smoking status (yes)	1 (4.5)	3 (27)	1 (7.7)
Calcium (mmol/L)	2.36 [2.29-2.40]	2.34 [2.30-2.41]	2.30 [2.28-2.36]
Phosphate (mmol/L)	1.12 [1.03-1.23]	1.25 [0.89-1.33]	1.24 [1.14-1.27]
25OH-vitamin D (nmol/L)	77.2 [49.0-84.9]	51.6 [40.4-62.6]	52.6 [40.7-72.2]
CTX (ng/mL)	0.592 [0.427-0.729]	0.373 [0.288-0.513]	0.514 [0.411-0.618]
P1NP (ng/mL)	76.8 [53.8-87.2]	64.3 [48.7-77.7]	65.5 [55.7-72.7]

Numerical variables are presented as median [interquartile range]; categorical variables as n (%).

Abbreviations: BMI, body mass index; CTX, C-terminal telopeptide of type I collagen; P1NP, N-terminal propeptide of type I collagen.

^*a*^6 patients with isolated AVP deficiency and 5 patients with panhypopituitarism.

### Calcium-phosphate parameters and bone turnover markers

Serum levels of calcium, phosphate, and 25OH-vitamin D across the 3 study groups are presented in Table 2. No significant differences were observed regarding calcium and phosphate, whereas 25OH-vitamin D levels were slightly lower in patients with AVP-D compared to HC (*P* = .031); no difference was observed between patients with AVP-D and PP (*P* = .733).

**Figure 1:**
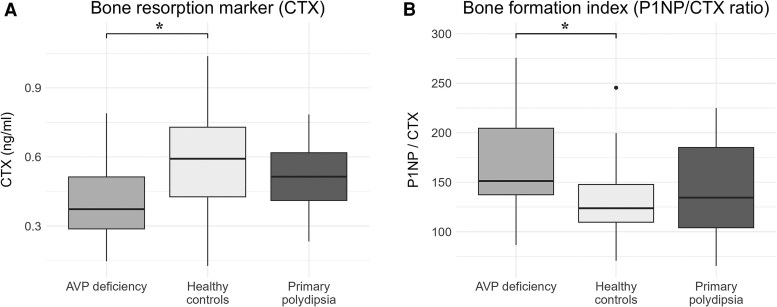
Levels of C-terminal telopeptide of type I collagen (CTX, A) and the bone formation index (P1NP/CTX, B) in patients with arginine vasopressin (AVP) deficiency, primary polydipsia, and in healthy controls. P1NP, N-terminal propeptide of type I collagen. *P < .05 for the comparison between patients with AVP deficiency and healthy controls.

Regarding bone turnover markers, patients with AVP-D exhibited lower CTX levels compared to HC (0.373 [0.288-0.513] vs 0.592 [0.427-0.729] ng/mL, *P* = .021), whereas no significant difference was observed when compared to individuals with PP (0.514 [0.411-0.618] ng/mL, *P* = .134) (Table 2, Fig. 1A). This difference in CTX levels remained significant after excluding the patient treated with budesonide (*P* = .031). Consistent results were also observed when separately analyzing patients with panhypopituitarism (0.373 [0.303-0.378] ng/mL, *P* = .028), whereas only a tendency was observed regarding patients with isolated AVP-D (0.397 [0.278-0.608] ng/mL, *P* = .175). The reduction in CTX levels in patients with AVP-D compared to HC was also confirmed in a linear regression model adjusted for age, sex, and smoking status (β = −0.183; 95% CI, −0.352 to −0.013; *P* = .036) (Table 3).

**Table 3:** Results of linear regression models with C-terminal telopeptide of type I collagen (CTX) as a dependent variable using healthy controls as the reference group

	Model 1 (Diagnosis), β (95% CI), *P* value	Model 2 (+ Age), β (95% CI), *P* value	Model 3 (+ Sex), β (95% CI), *P* value	Model 4 (+ Smoking status), β (95% CI), *P* value
**Independent variable**				
Intercept	0.606 (0.514-0.697) *P* < .001	0.689 (0.517-0.861) *P* < .001	0.724 (0.537-0.910) *P* < .001	0.722 (0.529-0.915) *P* < .001
AVP deficiency	−0.193 (−0.351 to −0.036) *P* = .018	−0.180 (−0.339 to −0.021) *P* = .027	−0.181 (−0.340 to −0.021) *P* = .027	−0.183 (−0.352 to −0.013) *P* = .036
Primary polydipsia	−0.079 (−0.229 to −0.070) *P* = .292	−0.066 (−0.217 to 0.085) *P* = .382	−0.046 (−0.203 to 0.110) *P* = .553	−0.047 (−0.206 to 0.112) *P* = .554
Age	—	−0.003 (−0.007 to 0.002) *P* = .255	−0.003 (−0.007 to 0.002) *P* = .263	−0.003 (−0.007 to 0.002) *P* = .277
Sex (female)	—	—	−0.066 (−0.202 to 0.070) *P* = .332	−0.065 (−0.205 to 0.074) *P* = .351
Smoking status (yes)	—	—	—	0.009 (−0.211 to 0.228) *P* = .936

Abbreviation: AVP, arginine vasopressin.

In contrast, no differences were found in P1NP levels among the 3 groups (AVP-D: 64.3 [48.7-77.7] ng/mL; PP: 65.5 [55.7-72.7] ng/mL; HC: 76.8 [53.8-87.2] ng/mL) (Table 2). Similarly, no association emerged in the regression model adjusted for diagnosis, age, sex, or smoking status (β = −9.114; 95% CI, −26.553 to 8.324; *P* = .297).

The bone formation index (P1NP/CTX ratio) was higher in patients with AVP-D (151 [137-204]) compared to HC (124 [110-148], *P* = .036), whereas no difference was observed between patients with PP (134 [104-185]) and either group (lowest *P* value: PP vs AVP-D, *P* = .252) (Fig. 1B). In the multivariable regression model adjusted for age and sex, AVP-D remained significantly associated with a higher P1NP/CTX ratio compared to HC (β = 36.8; 95% CI, 0.9-72.7; *P* = .045). However, significance was marginally lost when additionally adjusting for smoking status (β = 37.7; 95% CI, −0.6 to 75.9; *P* = .053) (Table 4). No significant differences were found between patients with PP and either group.

**Table 4:** Results of linear regression models with bone formation index (P1NP/CTX ratio) as a dependent variable using healthy controls as the reference group

	Model 1 (Diagnosis), β (95% CI), *P* value	Model 2 (+ Age), β (95% CI), *P* value	Model 3 (+ Sex), β (95% CI), *P* value	Model 4 (+ Smoking status), β (95% CI), *P* value
**Independent variable**				
Intercept	131.3 (111.2-151.5) *P* < .001	119.6 (81.2-157.9), *P* < .001	121.9 (79.9-163.9), *P* < .001	122.6 (79.0-166.2), *P* < .001
AVP deficiency	38.7 (3.8-73.5), *P* = .031	36.8 (1.4-72.3), *P* = .042	36.8 (0.9-72.7), *P* = .045	37.7 (−0.6 to 75.9), *P* = .053
Primary polydipsia	8.8 (−24.3 to 41.8), *P* = .595	6.9 (−26.7 to 40.6), *P* = .680	8.2 (−27.0 to 43.5), *P* = .640	8.5 (−27.4 to 44.4), *P* = .634
Age	—	0.36 (−0.64 to 1.37), *P* = .470	0.37 (−0.65 to 1.38), *P* = .472	0.35 (−0.68 to 1.39), *P* = .493
Sex (female)	—	—	−4.4 (−35.0 to 26.2), *P* = .774	−4.8 (−36.2 to 26.7), *P* = .761
Smoking status (yes)	—	—	—	−3.67 (−53.09 to 45.75), *P* = .882

Abbreviations: AVP, arginine vasopressin; CTX, C-terminal telopeptide of type I collagen; P1NP, N-terminal propeptide of type I collagen.

## Discussion

Our study shows that patients with AVP-D exhibit reduced CTX levels compared to healthy controls but not compared to patients with PP. In contrast, bone formation, as reflected by P1NP concentrations, was comparable across the 3 groups. As a consequence, the bone formation index (P1NP/CTX ratio) was higher in patients with AVP-D compared to healthy controls, but showed no difference when compared to PP. Taken together, our findings are not suggestive of relevant alterations in bone metabolism with potential detrimental effects in patients with AVP-D.

The role of both AVP and OXT in bone metabolism has been increasingly recognized over the past decades. In 2013, Tamma et al provided compelling in vitro and in vivo evidence for the catabolic effects of AVP on bone (4). These effects were shown to be mainly mediated through activation of the V1a receptor and downstream Erk signaling, leading to decreased bone formation by osteoblasts and enhanced resorptive activity by osteoclasts. In contrast, stimulation of the V2 receptor appeared to have a negligible impact on bone homeostasis. Consistent with these findings, mice lacking the V1a receptor or treated with AVP antagonists exhibited a profound increase in bone mass (4).

The same research group had previously demonstrated that OXT exerts anabolic effects on bone: deletion of the hormone or its receptor led to osteoporosis in mice, due to impaired bone formation (19). However, in 2016, Sun et al showed that mice lacking both AVP and OXT (double mutants) exhibited only mildly increased bone mass (5). These findings suggest that among the 2 neurohypophyseal hormones, AVP likely exerts the predominant skeletal effect, as the combined deficiency results in a phenotype of increased bone mass due to suppressed resorption, rather than bone loss from impaired formation.

Despite these strong physiological foundations, human data on the deficiency of AVP and possibly of OXT are scarce and inconclusive. In 1998, Pivonello et al reported a reduction in BMD at both the lumbar spine and femoral neck in a cohort of young patients with AVP-D, compared to age- and sex-matched healthy controls. Interestingly, this bone loss was attributed to reduced bone formation, as indicated by decreased serum osteocalcin levels, whereas bone resorption—assessed by urinary NTX—remained within the normal range (9).

However, large-scale data from the subsequent study by Wüster et al, based on the Pharmacia & Upjohn International Metabolic Database, failed to demonstrate BMD differences between patients with and without AVP-D. Nonetheless, an increased fracture risk was observed in male patients (20). Since this finding could not be fully explained by decreased BMD, other factors must be considered. These may include coexisting pituitary hormone deficiencies—either untreated or inadequately treated—that can impair bone integrity (21, 22). Additionally, an increased risk of falls could be involved, potentially resulting from visual impairment or hyponatremia resulting from desmopressin treatment (20, 23, 24), with hyponatremia itself being known to also exert a direct detrimental effect on bone (25-27).

In 2021, Aulinas et al evaluated BMD and hip structural analysis in 17 male patients with AVP-D compared to 20 males with isolated anterior pituitary dysfunction (11). Although mean BMD and hip structural analysis parameters did not differ significantly between the 2 groups, patients with fasting OXT levels below the median of the population displayed significantly lower BMD *Z*-scores at all measured sites. Moreover, in patients with AVP-D—but not in those with anterior pituitary dysfunction—fasting OXT levels were strongly and positively associated with favorable hip geometry and strength indices at the intertrochanteric region. In contrast, no associations were found between bone parameters and AVP levels in either group (11).

Altogether, the limited evidence currently available seems to suggest a potentially deleterious effect of AVP-D on bone metabolism, with likely reduced bone mass in these patients. Our study, however, aligns more closely with preclinical findings than with previous clinical data, suggesting a possible reduction in bone resorption in these patients. In our cohort, CTX levels were mildly reduced in patients with AVP-D compared to healthy controls, whereas patients with PP were in between and showed no significant differences from either group. Similarly, the bone formation index was higher in patients with AVP-D compared to healthy controls, whereas no significant differences were observed between individuals with PP and the other 2 groups.

These findings warrant a few considerations. Although our data are insufficient to support a clear effect of the lack of AVP on bone metabolism, interestingly, patients with PP exhibited median CTX levels that tended to be lower than those of healthy controls, albeit without statistical significance. Therefore, it is possible that a larger sample size of patients with PP would have shown a significant difference. At the same time, the organic deficiency of AVP observed in patients with AVP-D could have a greater impact on bone metabolism than the fluctuating suppression of AVP levels in patients with PP. Patients with AVP-D entirely lack the V1a receptor-mediated resorptive stimulus, whereas in PP, some degree of receptor activation is likely preserved, resulting in an intermediate effect between the other 2 groups.

An alternative explanation for the reduction in CTX observed in patients with AVP-D may lie in the higher prevalence of smokers in this group, as smoking has been reported to reduce bone turnover markers (28, 29). However, all models were adjusted for smoking status, and although the association between AVP-D and the bone formation index was slightly reduced, the association with lower CTX remained statistically significant. Additional factors may account for discrepancies with prior clinical studies, particularly the study by Pivonello et al, the only one reporting bone turnover marker data (9). Unlike that study, we measured both CTX and P1NP, which are currently the bone turnover markers most widely recommended by international scientific societies, including the International Osteoporosis Foundation and the International Federation of Clinical Chemistry (30, 31). Although serum CTX and urinary NTX are considered comparably reliable as markers of bone resorption, P1NP is generally regarded as a more accurate and stable marker of bone formation compared to osteocalcin, as it has a significantly longer half-life and reduced circadian variation (32, 33). Furthermore, information regarding 25OH-vitamin D levels was not reported in that cohort. In vitamin D-deficient patients undergoing supplementation, reduced bone turnover has been described, particularly with a decrease in P1NP levels (34). Therefore, it is possible that some of the patients with AVP-D in that cohort, also in consideration of impaired BMD, were receiving vitamin D supplementation, potentially contributing to the observed findings. In contrast, in our cohort, no patient with AVP-D was receiving vitamin D supplementation. Of note, patients with AVP-D in our study had slightly lower 25OH-vitamin D concentrations than healthy controls. As vitamin D deficiency is generally associated with higher bone resorption markers (35, 36) or, in some cases, with a neutral effect (34, 37, 38), the observation of lower CTX levels in patients with AVP-D despite reduced vitamin D concentrations may further support the finding of a reduction in bone resorption in this population.

Regarding the potential coexistence of OXT deficiency in patients with AVP-D, one might expect a reduction in bone formation markers; however, this was not observed in our study. Preclinical studies using murine and human osteoblast models have shown that, in addition to its hypothalamic origin, OXT is also locally synthesized in the bone marrow and that this process is stimulated by estrogens (39-41). Locally produced OXT acts in both autocrine and paracrine fashion on osteoblasts, promoting their proliferation and enhancing bone formation (39). Notably, this local OXT production appears to be more critical for skeletal homeostasis than centrally derived OXT (3, 42, 43). Supporting this, central administration of OXT in OXT-knockout mice failed to affect bone turnover or osteoblast and osteoclast differentiation, whereas peripheral OXT administration in ovariectomized rats prevented osteoblast and osteocyte loss and attenuated the rise in bone turnover markers (19, 43). These findings support the hypothesis that, even in the presence of possible central OXT deficiency, patients with AVP-D may retain normal bone formation because of preserved peripheral OXT production, thereby preventing a significant reduction in bone formation markers.

Our study presents some strengths and limitations. A major strength lies in the inclusion not only of healthy individuals but also of patients with PP, who are likely to have chronically reduced AVP levels resulting from excessive water intake while preserving normal OXT secretion. This unique comparison group allowed us to better disentangle the isolated effects of the lack of AVP from those potentially attributable to other factors. Although no statistically significant differences were observed between the PP group and the other 2 groups, the inclusion of PP patients partly mitigates the risk of overattributing the observed changes in bone turnover to AVP-D alone. Without this group, our findings might have suggested a more direct and evident role of AVP in bone metabolism. Furthermore, unlike previous studies, we assessed both CTX and P1NP levels, which are currently considered by leading international scientific societies as among the most sensitive and specific bone turnover markers, and that were collected in accordance with international recommendations (30, 31): in the morning, under fasting conditions, and in patients who had not engaged in intense physical activity during the previous 24 hours.

Some limitations must also be acknowledged. The most relevant limitation is the lack of data on BMD, as only bone turnover markers were available. Consequently, it was not possible to integrate the biochemical findings with structural bone outcomes across the 3 groups. Moreover, this was a cross-sectional study, and because of the rarity of the condition, the sample size was relatively small, which may have affected our results. Finally, some differences could potentially be related to the presence of isolated AVP-D vs panhypopituitarism. However, because of the limited cohort size, stratification by etiology could not be reliably evaluated, and the potential effects of concomitant hormone replacement therapy could not be fully ruled out. Nonetheless, all patients with additional hormonal deficits were on stable replacement therapy.

## Conclusion

In conclusion, our study showed that patients with AVP-D had lower CTX levels, a marker of bone resorption, than healthy controls, whereas P1NP concentrations, which reflect bone formation, were similar across all 3 groups. As a consequence, the P1NP/CTX ratio was higher in patients with AVP-D than in HCs, whereas this ratio in individuals with PP did not differ from either of the other 2 groups.

Although the absence of AVP in patients with AVP-D could theoretically lead to reduced bone resorption, the observation that CTX levels in patients with PP—who similarly exhibit chronically suppressed or fluctuating AVP levels due to excessive fluid intake—were comparable to those of HCs does not allow for a definitive conclusion regarding the impact of the lack of AVP on bone metabolism in humans. Nonetheless, if such an effect exists, contrary to previous reports, it does not appear to be detrimental.

Further large-scale, prospective studies integrating both bone turnover markers and BMD assessments are warranted to confirm and expand on these findings.

## Data Availability

This study is a secondary analysis of data from a previously published clinical trial registered on ClinicalTrials.gov (NCT05890690). No new datasets were generated or deposited. We may share deidentified, individual participant-level data that underlie the results reported in this article and related documents, including the study protocol. All requests should be sent to the corresponding author. The steering committee of this study will discuss all requests and decide, based on the scientific rigor of the proposal, whether data sharing is appropriate. All applicants are asked to sign a data access agreement.

## Abbreviations

AVP: arginine vasopressin
AVP-D: arginine vasopressin deficiency
BMD: bone mineral density
CTX: C-terminal telopeptide of type I collagen
HC: healthy control
NTX: N-telopeptide of type I collagen
OXT: oxytocin
P1NP: N-terminal propeptide of type I collagen
PP: primary polydipsia
